# Primate comparative neuroscience using magnetic resonance imaging: promises and challenges

**DOI:** 10.3389/fnins.2014.00298

**Published:** 2014-10-06

**Authors:** Rogier B. Mars, Franz-Xaver Neubert, Lennart Verhagen, Jérôme Sallet, Karla L. Miller, Robin I. M. Dunbar, Robert A. Barton

**Affiliations:** ^1^Department of Experimental Psychology, University of OxfordOxford, UK; ^2^Nuffield Department of Clinical Neurosciences, Centre for Functional MRI of the Brain, University of Oxford, John Radcliffe HospitalOxford, UK; ^3^Donders Institute for Brain, Cognition and Behaviour, Radboud University NijmegenNijmegen, Netherlands; ^4^Department of Anthropology, Durham UniversityDurham, UK

**Keywords:** neuroecology, MRI, diffusion MRI, connectivity, phylogenetics

## Abstract

Primate comparative anatomy is an established field that has made rich and substantial contributions to neuroscience. However, the labor-intensive techniques employed mean that most comparisons are often based on a small number of species, which limits the conclusions that can be drawn. In this review we explore how new developments in magnetic resonance imaging have the potential to apply comparative neuroscience to a much wider range of species, allowing it to realize an even greater potential. We discuss (1) new advances in the types of data that can be acquired, (2) novel methods for extracting meaningful measures from such data that can be compared between species, and (3) methods to analyse these measures within a phylogenetic framework. Together these developments will allow researchers to characterize the relationship between different brains, the ecological niche they occupy, and the behavior they produce in more detail than ever before.

Humans have an unusually large brain, comprising about 90 billion neurons connected by many trillions of synapses, organized into systems of staggering complexity. This elaborate neural network is pivotal to what makes humans unique, and comprises some of the most compelling open questions in science: What is it all for, and how does it work? Embedded in these questions is an assumption that neural structures and mechanisms are products of a design process, albeit a blind one: the process of evolution by natural selection. To understand how the brain works, we need to know more about what problems the brain evolved to solve and how these relate to its organization. Questions about brain function, organization, and evolution of the brain are therefore complementary.

Comparisons between species are an essential tool in answering evolutionary questions about brain organization. In fact, only by understanding more about other primates' brains can we understand the uniqueness of our human brain. Comparative neuroscience often focuses on a limited set of “model” species, aiming to study aspects of brain function that are conserved across species. Within this approach, species differences are merely inconvenient confounds. However, the comparative paradigm has much greater potential when it exploits rather than ignores differences between species (Preuss, 2000). Primate brains differ in aspects of structural detail as well as in overall size (Rilling, 2006)—implying specific adaptations, driven by the ecological niche the species are living in and their particular evolutionary history. In the ideal case, all these factors are considered leading to a full account of variations in brain organization and behavioral repertoire across the primate order. The discovery of general neuro-evolutionary principles can then be used to place the features of particular species such as humans in broader context. The goal of such a “neuroecological” comparative approach is thus to understand the general design principles and constraints dictating the evolution of neural systems, and the selection pressures that caused species differences in these systems. Importantly, evolutionary processes and patterns can only be rigorously identified by investigating a large range of species (Striedter, 2005).

The success of the neuroecological approach thus requires phylogenetically broader comparisons than is typical in neuroscience. In this paper, we explore the feasibility of meeting this requirement. We will argue that recent advances in magnetic resonance imaging technology and data analyses mean that goal can now be realized to a much greater extent than was previously possible. We will thus focus in this review on recent developments in methodology (Figure 1); we reserve a more extensive discussion of empirical discoveries for future communications.

**Figure 1:**
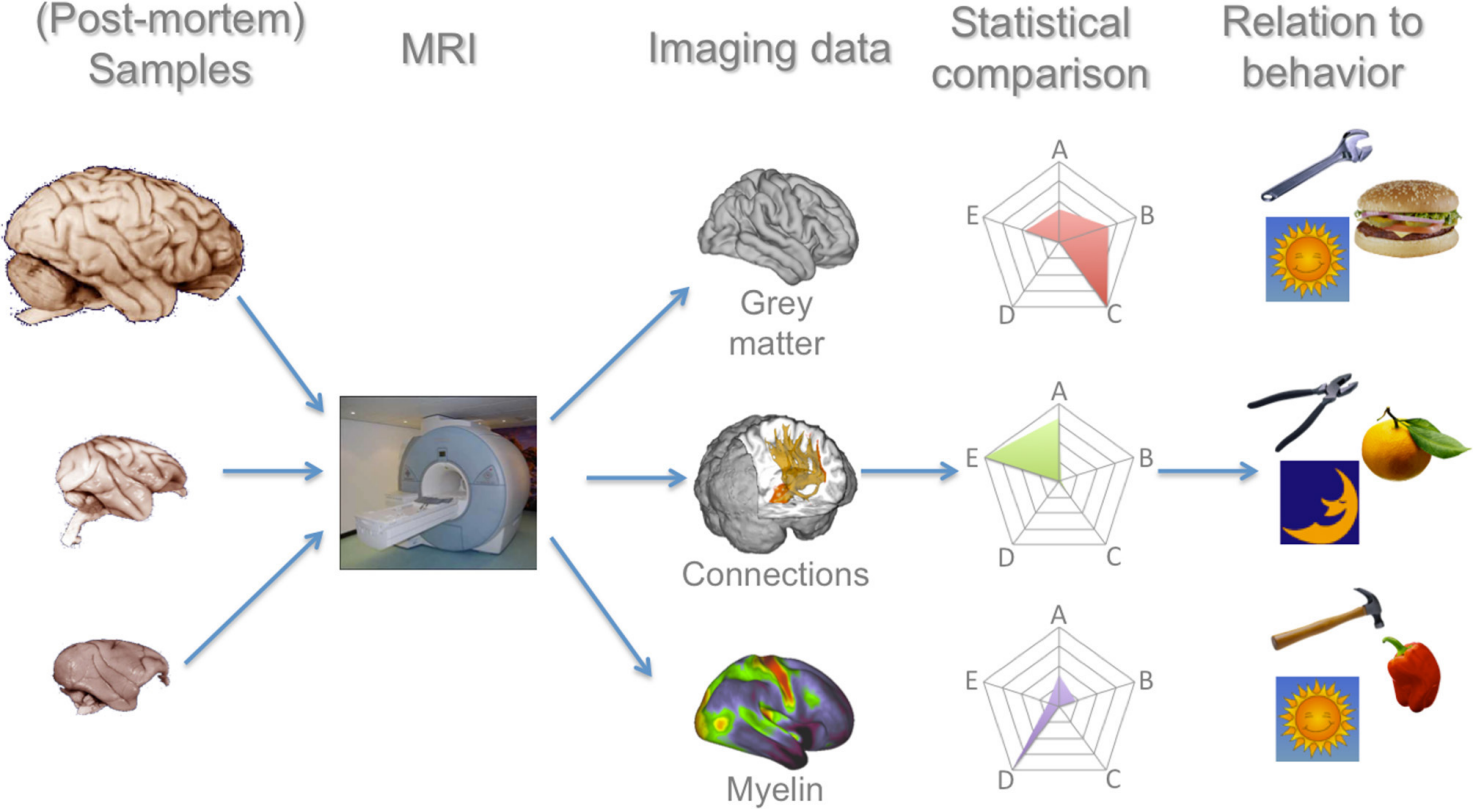
**Processing pipeline for a comparative primate neuroimaging research program**. In this manuscript we argue that each of these steps have seen recent advances that now allow such a program to be realistically feasible. MRI of whole-brain (post-mortem) samples allows a number of measures to be collected, for which comparative analysis techniques have now been developed and validated. These data can then be related to the large body of ecological data on these species. Brain images in the left column are reproduced from the University of Wisconsin and Michigan State Comparative Mammalian Brain Collections and the National Museum of Health and Medicine (www.brainmuseum.org); preparation of these images was funded by the National Science Foundation and National Institutes of Health.

## Variables of interest in comparative neuroscience

When investigating patterns of brain organization across a large range of species, one would ideally want to study aspects of brain organization that have plausible behavioral correlates and that allow an explanation of their causes. Among the aspects of brain organization that have been proposed to be of interest for this endeavor are the size of the brain, the number, relative size, and location of specific cortical fields, and the connections between different brain areas (Krubitzer and Kaas, 2005). Comparative neuroscience has at its disposal an arsenal of tools to study each of these different aspects in great detail. For instance, in order to determine the location and size of individual brain regions in different brains, it is possible to study microscopic features of brain organization such as cytoarchitecture, myelinization, and chemoarchitecture (Zilles et al., 2002). All of these features have been shown to be reliable indicators when trying to identify individual brain regions. The architecture of connections between different brain areas can be studied in tract tracing studies. Tracers are substances or viruses that are injected in a certain part of the brain that then travel along axonal pathways between areas, providing an extremely detailed picture of the connections of different areas (Morecraft et al., 2009). These methods present the “gold standard” in comparing brain structure between species and they are continuously being refined (e.g., Markov et al., 2014).

Although each of these methods yields very detailed information about the tissue under investigation they are often very laborious and expensive to use. In the case of tracer studies, for example, each area of investigation requires a new subject to be injected and, after a time delay for the tracer to travel through the brain, to be sacrificed and the brain sliced up and investigated. The results of different investigations can be combined together in large databases allowing researchers to systematically compare results across the whole brain (Bakker et al., 2012), but this has in fact been done only for a few species. Although some of the pioneers of neuroanatomy already focused on comparisons between species (Brodmann, 1909) and recent studies have continued this work using the various tools available (e.g., Preuss and Goldman-Rakic, 1991; Schenker et al., 2005), the costs and time investment required mean that this approach is not often applied to more than four or five species. Therefore, in parallel to this work, researchers have searched for indices of brain organization that can be studied more easily in a larger range of species.

In the absence of information on microscopic features in most primate species brains, studies on variation in brain organization have predominantly focused on derived global measures such as the size of the brain relative to the whole body or the size of the neocortex relative to an area of the brain that is hypothesized not to show species-specific changes, such as the brainstem, or on the size of large, easily identifiable subsections of the brain. Unfortunately, invaluable as these measures are, they cannot but obscure much of the interesting data (Healy and Rowe, 2007; Barton, 2012). Whole brain size is likely to be a result of various changes at lower levels of description, such as the macro level of brain areas and brain systems. Changes at the lower level, such as in the size and connections of individual brain areas, might more plausibly be related to specific behavioral variables, as has been shown for social cognition (Sallet et al., 2011) and tool use (Quallo et al., 2009).

Studies focusing on the size of individual brain areas have had to rely on macroscopic anatomical features such as sulcal anatomy or other clearly identifiable boundaries to demarcate an area. Although this method has proven successful for subcortical areas (Barton and Harvey, 2000; Barton, 2006; Balsters et al., 2010), in the cortex these markers often bear little relationship to the borders between areas as established using microscopic features such as cytoarchitecture (Amunts et al., 2007). Moreover, comparison of different cortical sulci between species can be misleading. For instance, the human cerebral cortex has a sulcus labeled “superior temporal sulcus” (STS), similarly to a label used in the macaque, but the dramatic expansion and reorganization of the macaque temporal cortex (Van Essen and Dierker, 2007) challenges the assumption that the STS is completely homologous between the two species. In the case of the prefrontal cortex these problems are amongst those that have contributed to a long-standing debate on whether the prefrontal cortex is especially large in humans compared to other primates, whether it is especially large in all great apes compared to other primates, or whether prefrontal cortex size follows the pattern predicted by allometric scaling (Barton and Venditti, 2013).

Even more substantial is the problem that the size of individual brain areas itself may be a misleading measure. It is now well appreciated that the picture of selective expansion of one brain area while others remain constant is simplistic. A more likely scenario is the correlated expansion of connected areas (Barton and Harvey, 2000). This requires one to study the size of many brain regions simultaneously, which is very laborious using most conventional research techniques. Moreover, the volume of a brain area might not necessarily reflect changes in the computational aspects of the brain, such as the number of neurons or the number of connections between neurons (Herculano-Houzel, 2011). Finally, and perhaps most fundamental, while similar areas might be present in different species, other aspects of their organization, such as their connections, might very well differ. A change in the connectional architecture of the brain, for instance, can have substantial influence on the information that such regions have access to (Rilling et al., 2008). Such changes would not be identified by conventional methods comparing the size of brain areas or even white matter volume between species.

For these reasons, the large-scale comparative approach necessitates that we are able to study more detailed aspects of brain organization. Moreover, if this approach is to yield fruitful results, we need to be able to acquire such data quickly and reliably in a large number of species. As we will argue below, recent advances might just make this possible.

## Advances in data acquisition

As discussed above, a major obstacle for large comparative investigations is that most traditional research methods are very labor-intensive. Even with the development of automated methods, such as seen in recent cytoarchitectonic and receptor mapping studies (Schleicher et al., 1999), such studies are still arduous and can only be applied to a limited part of the cortex at any one time. The establishment of magnetic resonance imaging (MRI) as a primary tool for cognitive neuroscience has the potential to alleviate this problem. MRI allows one to collect whole-brain, quantitative images within a relatively short time frame. Standard protocols to acquire a structural scan used to map the gray matter architecture of the whole brain can be acquired in a few minutes and the acquisition time of even the most high-resolution scans is measured in hours. This does not mean the analysis time of imaging data is not substantial, but the ability to acquire whole-brain data within a short time span presents a substantial advantage.

Different MRI techniques are now available that allow it to be used as a full-scale neuroanatomical technique and many of these techniques are now starting to be used for comparative research, as recently reviewed by Rilling (2014). Early studies used structural MRI data to obtain data on the size of the brain and its subdivisions. This work showed that the different primate brains are not simply scaled-up versions of one another, but that there are idiosyncratic expansions of different parts of the cortex in different primate species (Rilling, 2006). The ever increasing ability to obtain better contrast and higher resolution structural scans means that quality of the data obtained in structural studies can be expected to continue to improve well into the future. However, although this approach allows one to collect data of a wide range of species, it still suffers from some of the problems outlined above when defining separate areas based on macroscopic landmarks.

MRI cannot currently be used to directly quantify the microscopic features of brain organization used in traditional comparative neuroscience. MRI is broadly sensitive to a wide range of cytoarchitectonic variations, and this lack of specificity makes it difficult to relate a given signal pattern to a particular aspect of the microstructure. These problems not withstanding, in recent years various groups have tried to exploit the differential sensitivity of different MRI sequences to various tissue properties to approximate those properties on a voxel-by-voxel basis (i.e., in each three-dimensional pixel of the brain). For example, a number of recent studies exploit T1-weighted imaging to demonstrate myelin-rich areas, which can be used to define specific parts of the visual cortex (Barbier et al., 2002) or changes in myeloarchitecture across the cortex (Bock et al., 2009). Using the ratio of T1-weighted and T2-weighted images in an attempt to improve the signal-to-noise ratio Glasser and Van Essen sought to illustrate the distribution of myelin across the cortex (Glasser and Van Essen, 2011), showing good correspondence with known myelinization of the cortical gray matter. Further demonstrating the potential of such approaches, two recent studies using retinotopy and tonotopy showed that they are capable of reliably identifying distinct visual and auditory areas based on myeloarchitecture (Dick et al., 2012; Sereno et al., 2013). Glasser and colleagues compared the distribution of cortical myelin between humans, chimpanzees, and macaques, illustrating the potential of this approach for comparative studies (Glasser et al., 2014). These works demonstrate the possibility of using MRI as a comparative tool. The recent development of protocols quantifying a number of different parameters together with myelin (Haacke et al., 2005; Laule et al., 2007; Weiskopf et al., 2013) holds the promise of extending this approach to various other tissue properties.

Apart from structural gray matter imaging, the MRI techniques that are most often used in comparative neuroscience look at connectivity between areas of the brain. Diffusion MRI allows the quantification of the diffusion of water molecules in the brain. Since water diffusion is generally least constrained along the axis of white matter fibers, this information can be used to obtain a quantitative image of the brain's white matter architecture (Johansen-Berg and Rushworth, 2009). Using tractography algorithms it is then possible to follow the path of specific white matter fiber bundles, providing an index of the connections between particular areas and the routes these connections take. Although tractography is an indirect measure and is not without its limitations (Jbabdi and Johansen-Berg, 2011), it has been applied successfully in a number of comparative studies (reviewed in Rushworth et al., 2014). A number of recent studies have combined diffusion MRI with another indirect measure of neural connectivity, looking at spontaneous brain activity during rest, so-called resting state functional MRI (rs-fMRI). This technique relies on the correlation of signal variations in time across different parts of the brain when animals are not engaged in any task. These correlations, termed functional connectivity, have been shown to be at least partly dependent on structural connections (O'Reilly et al., 2013)—although these connections need not be monosynaptic (Honey et al., 2009)—and generally identify networks of areas that tend to co-activate during task performance (Smith et al., 2009). These networks can even be identified when an animal is under light anesthesia (Vincent et al., 2007). Recent work using this technique showed that rs-fMRI can reliably identify functional connectivity between areas that are known to be structurally connected and that it can be used as a comparative measure between humans and macaques (Margulies et al., 2009; Mars et al., 2011; Hutchison and Everling, 2013; Birn et al., 2014).

MRI-based techniques thus offer a tremendous potential for acquiring various measurements across whole brains within a reasonable time frame, offering opportunities for full-scale comparative neuroanatomy. However, neuroimaging techniques are not without their disadvantages. First, the signals obtained are indirect measures of tissue properties, for instance the diffusion of water molecules as a proxy for the orientation of a white matter fiber in the case of diffusion MRI and the differential sensitivity to tissue properties used to approximate myelin content. Second, MR imaging techniques currently do not have the very fine resolution that traditional anatomical techniques have, with diffusion MRI of 1 mm resolution and rs-MRI of 1.5 mm resolution now standard for a macaque-sized brain. However, neuroimaging is still a relatively young field and the resolution of MRI is being improved continuously.

A very different advantage of MRI-based techniques is that they are not destructive. Although that means the technique can potentially be used on living animals (indeed it has to be in the case of resting state fMRI), it yields a more general advantage. In traditional neuroanatomical research, if one wants to investigate the same area of the brain using different stains, adjacent slices have to be compared that each have been prepared using a different stain. In the context of MRI the same research question can be addressed simply by scanning the same tissue multiple times using different scanning protocols. Animals, however, still have to be scanned, which places a substantial limitation on which species can be studied. Most laboratory work on primates focuses on macaques and marmosets. Opportunities to scan live apes are extremely limited and are expected to become even more scarce in the near future (Rilling, 2014). Therefore, the most fruitful application of MRI in comparative studies might lie in post-mortem imaging. Several groups have focused on adapting the techniques described above for work on brain samples from individuals that have died of natural causes. Successful results have been obtained using structural imaging, myelin mapping, and diffusion MRI (McNab et al., 2009; Geyer et al., 2011), which can potentially all be obtained from the same samples. This approach has tremendous potential for researchers collecting brain samples from animals that have died in zoos and research institutes of causes unrelated to their research and provide a scientific use of cadavers beyond the natural life of the animal.

## Advances in data analysis

### Identifying and comparing areas

With the increasing availability of comparative MRI data, the next requirement is to develop analysis methods that match the complexity and richness of these data. Before discussing approaches for comparative anatomy that can only be achieved using MRI, we will first explore the use of MRI data to mimic the goals of the traditional anatomical research techniques in identifying distinct brain regions that can then be compared across species. Various approaches have been put forward to this end. A relatively straightforward approach is to use an algorithm that searches for distinct changes in a given property between voxels. For instance, Cohen and colleagues used the change in whole-brain functional connectivity to identify the border between functional areas in the human neocortex (Cohen et al., 2008). Glasser and Van Essen used a similar procedure to identify the gradient of change in cortical myelin content to demonstrate the border between areas (Glasser and Van Essen, 2011). In histological work, some groups use a combination of different stains to identify areas (Carmichael and Price, 1994) and this approach could potentially be mimicked by using data obtained with different MRI protocols (Weiskopf et al., 2013).

**Figure 2:**
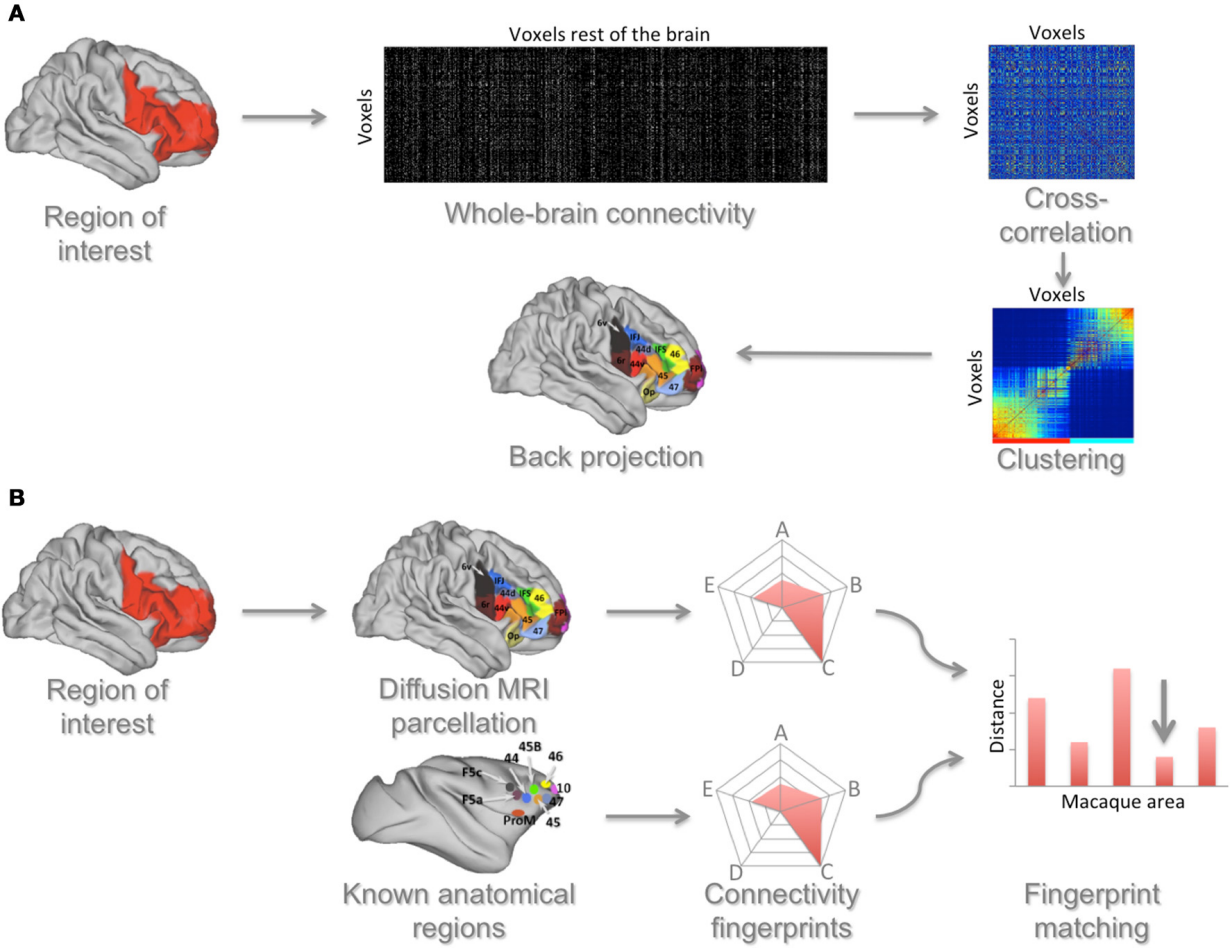
**Connectivity-based approach to identifying and comparing brain areas**. **(A)** A region of interest (ROI) is defined and the connectivity of each voxel in this region with each voxel in the rest of the brain is determined. From these data a cross-correlation matrix is calculated that indicates each ROI voxel's similarity in connections to each other ROI voxel. This matrix is then reordered to group together voxels that have the most similar connectivity profiles. This reordered matrix is then backprojected onto the brain, identifying connectivity-based clusters. Based on Neubert et al. (2014). **(B)** These areas can then be matched to areas in the macaque brain. In this approach the connectivity of human areas, as established using the approach described in **(A)**, with selected areas in the rest of the brain is determined. The same is done for cytoarchitectonically defined areas in the macaque brain. These connectivities are summarized in a spider plot showing the connectivity of a given area with areas that have known homologs between species. A distance measure between the human area and all macaque areas is then calculated, showing the area that has the most similar connectivity between species (indicated by the arrow in the figure).

An alternative to searching for borders is to employ a clustering algorithm that groups together voxels based on their similarity (Figure 2A). Johansen-Berg et al. (2004) first demonstrated this approach using similarity in structural connectivity as established using diffusion MRI to identify brain areas. They showed that voxels on the medial surface of the human brain could be grouped together into two clusters based on their connectivity with the rest of the brain. The border between these clusters was consistent with the known anatomical border between the supplementary and pre-supplementary motor areas. Moreover, tasks designed to specifically activate these areas elicited functional neuroimaging responses in precisely these two clusters. Although this technique does not rely on microscopic features such as cytoarchitecture, the areas identified using this technique are meaningful anatomical units and often overlap with cytoarchitectonic areas (Caspers et al., 2011; Mars et al., 2011). The critical point of this approach is that it allows the identification of units of operation based on anatomical evidence without the need for task-based functional neuroimaging, making it ideal for comparative investigations. Illustrating its potential, recent work has used diffusion MRI to cluster parts of the human lateral parietal (Mars et al., 2011) and dorsal frontal (Sallet et al., 2013) association cortex and compared the resulting areas with known anatomical subdivisions in the macaque monkey.

The comparison of brain areas identified using one of these MRI-based techniques with areas in other species can be done in a quantitative manner (Figure 2B). Sallet et al. (2013) and Neubert et al. (2014) used diffusion MRI to parcellate the dorsal and ventral parts of the human frontal cortex and subsequently used rs-fMRI to identify the functional connectivity of each cluster with a number of cortical areas, defining a “connectivity fingerprint” (Passingham et al., 2002) of each cluster. Then, also using rs-fMRI, they created similar fingerprints for all prefrontal areas of the macaque monkey. A Manhattan distance measure was then used to match each human frontal area with each macaque frontal area. Most human areas were found to have a homolog in the macaque, but one cluster in the human lateral frontal pole could not be preferentially matched with any macaque area. This result dovetails with earlier suggestions that the lateral frontal pole is a uniquely human area (Koechlin, 2011) and with a recent independent identification of this area using cytoarchitectonic mapping (Bludau et al., 2014). Taking the approach of matching connectivity fingerprints even further, Mars and colleagues recently identified the unique connectivity fingerprint of an area in the human temporoparietal cortex associated with higher-order social cognition (Mars et al., 2012) and then searched for voxels with a similar connectivity profile across the entire macaque temporoparietal cortex, identifying the middle part of the macaque superior temporal sulcus as an anatomically homologous region (Mars et al., 2013).

### Beyond single areas

The techniques described above illustrate how MRI-based techniques can be used to identify specific areas in the brain and compare these across species. Given that MRI in general provides information about the entire brain, and thus can be used to acquire information about multiple areas at the same time, it can also be used to make inferences about the relationships between different areas. Again, this approach has its roots in traditional anatomical work. Kötter and colleagues used a database of work on macaque receptor density mapping and cortical connectivity and applied a hierarchical clustering analysis to demonstrate the relationship between areas in the macaque cortex (Kötter et al., 2001). This approach has been furthered by Averbeck and colleagues, who used the macaque connectivity database CoCoMac (www.cocomac.org) to assess the hierarchical relationship between areas of the frontal cortex and subsequently related these results to a similar analysis of parietal cortex, showing the existence of multiple, parallel parietal-frontal circuits (Averbeck and Seo, 2008; Averbeck et al., 2009). Such measures would be ideal to characterize differences in organization between brains. Again however, the sheer quantity of studies required to accumulate sufficient data for such analyses makes it all but impossible for traditional neuroanatomy. MRI-based measures allowing one to acquire information about multiple brain regions simultaneously make comparing such descriptives between species now feasible.

The hierarchical clustering approach moves beyond the comparison of single regions to potentially compare more complex aspects of brain organization. In fact, one of the most commonly used MRI protocols for comparative neuroscience focuses not on studying brain regions, but on comparing the white matter fiber pathways connecting parts of the brain. Such studies can identify differences in the relation different areas have to one another. In a now classic study, Rilling and colleagues compared the cortical projections of the arcuate fasciculus in humans, chimpanzees, and macaques (Rilling et al., 2008). They suggested that the arcuate has substantially expanded in the human brain, both when compared to the macaque and to the chimpanzee. This interpretation is compatible with the arcuate's proposed role in language, a uniquely human behavior. A number of recent studies have compared human diffusion MRI tractography with known pathways in the macaque obtained using traditional invasive tract tracing methods and suggested that other temporal-frontal pathways might also have expanded (Makris and Pandya, 2009), leading to a view of multiple dorsal and ventral temporal-frontal routes supporting language (Friederici and Gierhan, 2013). Caution is warranted, however, as most comparative connectivity studies rely on such a between-methods comparison, which means one cannot exclude the possibility that some of the reported differences are related to methodological differences. Work using the same method in two or indeed more species is at present still relatively rare. The increasing availability of post-mortem imaging holds the potential to change this.

Even when using MRI to study whole-brain anatomy of a large number of species, there are several important cautions that must be taken into account when comparing the results of MRI-based analyses between species. Most prevalent among these is that the comparison of brains of very different sizes means that one will often work with data of different resolutions compared to the brain. The use of post-mortem tissue can overcome some of the differences in acquisition resolution by allowing very long scans, but this still leaves some differences in scale in the analysis. Moreover, fixation of brains often results in shrinkage of the brain. To address some of these problems, the parameters of the tractography algorithms in comparative diffusion MRI studies are often adjusted to each particular brain and resolution (Jbabdi et al., 2013) and comparative statements often concern the relative size or connectivity of a set of areas compared to other areas in the same brain rather than comparing absolute numbers (Croxson et al., 2005; Neubert et al., 2014). In addition, a number of authors have taken the precaution of illustrating their methods on areas or connections that are hypothesized not to differ between species in order to demonstrate a baseline result in each species (Hecht et al., 2013). Another important problem concerns the interpretation of results. When searching for homologs between species (e.g., Mars et al., 2013; Sallet et al., 2013), caution is warranted, since algorithms searching for a “best match” between species will always yield a result. It is thus possible to label regions homologous between species even when this is clearly not the case. However, as discussed above, with caution the identification of areas in the human brain that have no homolog in the macaque is possible, as in the case of the lateral frontal pole discussed above (Neubert et al., 2014).

### Generating hypotheses

Most studies described above were only feasible because the authors had very strong anatomical hypotheses to test and could rely on a priori knowledge. For instance, to match connectivity fingerprints between species one needs to be able to identify homologous areas between species that can be used to determine the fingerprint. The studies comparing human and macaque brains described above only used regions for which the between-species homologs were known. Relatively few areas in the temporal cortex were used to define a connectivity fingerprint, since this part of the cortex has been suggested to have undergone a substantial reorganization since the last common human-macaque ancestor some 29 million years ago (Van Essen and Dierker, 2007). Indeed, Neubert and colleagues were able to show that the balance of functional connectivity between higher-order auditory areas and medial and lateral frontal cortex differs between these two species. In macaques, auditory association cortex shows preferential connectivity with areas in the cingulate cortex, whereas the homologous human area shows stronger functional connectivity with areas in the ventrolateral frontal cortex (Neubert et al., 2014). This result makes the auditory association cortex unsuitable as “reference” to compare other areas between species. This type of work thus requires substantial prior knowledge about the brains under investigation. However, when the number of species investigated increases it is inevitable that species will be studied whose neuroanatomy is mostly unknown. This will require the use of more data-driven approaches, at least in the first stages of research, to provide hypotheses and knowledge for subsequent, more detailed testing.

One important demonstration of a relatively unbiased comparison of primate brains using structural gray matter images was provided by Van Essen and Dierker (2007). These authors used surface-based registration to investigate which areas of a macaque brain need to be differentially expanded to morph it into a human brain. They showed that the middle part of the inferior parietal lobule, the temporal cortex, and frontal areas are most enlarged in the human as compared to the macaque brain. Although this approach does require some anatomical priors to identify anatomical markers that are presumed to be homologous, it provides a relatively unbiased identification of regions that researchers interested in differences between species could focus on. This approach was recently expanded to include the marmoset and capuchin monkey (Chaplin et al., 2013). Interestingly, this approach can also be applied to measures other than gray matter. Mantini and colleagues used the same technique to register networks of functional connectivity identified in macaques and human to one another (Mantini et al., 2013). They showed a parietal-frontal network in humans that could not be matched to any macaque network, consistent with observations of uniquely human parietal-frontal connectivity by Mars et al. (2011) and the observation that these areas have expanded disproportionately in humans (Van Essen and Dierker, 2007). Similar techniques were used by Hutchison et al. (2012) to study the functional connectivity of the frontal eye fields.

With the establishment of diffusion MRI and rs-fMRI and the launch of large-scale projects such as the Human Connectome Project (Van Essen et al., 2013), data mining of connectivity data has seen a spectacular growth. For rs-fMRI, independent component analysis (ICA) has been used as a data-driven tool for identifying networks of regions (Beckmann et al., 2005). Comparison of networks identified using ICA between species has now been reported for marmosets, baboons, macaques, and chimpanzees (Belcher et al., 2013; Wey et al., 2014). Similar approaches are being developed for diffusion MRI data as well (O'Muircheartaigh et al., 2011). Sporns and colleagues in particular have argued for the use of techniques developed in graph theory to study the organization of brain networks (Bullmore and Sporns, 2009). Such measures can provide a data-driven perspective on the organization of a brain. In the context of the human brain, these measures have suggested that the human brain is wired as a small world network, where a selected group of regions form “hubs” that are responsible for the majority of long-range connections (Van den Heuvel et al., 2012). Applying these measures to comparative diffusion MRI data, Li and colleagues suggested that hubs are largely conserved across macaques, chimpanzees, and humans (Li et al., 2013).

The clustering methods discussed in the previous sections can also be used in a more exploratory fashion. A number of studies have employed various statistical criteria to determine the number of clusters into which any part of the cortex can be divided (Kelly et al., 2010; Clos et al., 2013; Liu et al., 2013; Neubert et al., 2014). As such, clustering methods can be very useful as an exploratory tool, but they still invite a discussion of which criteria are able to identify the “correct” number of brain regions. Indeed, one can argue about the nature of what a brain region is. As acknowledged by the majority of these papers, the most important criterion is whether the solution is informative about the organization of these brains and can be used to compare different brains with one another. It is therefore important to keep in mind that an explorative approach is only the start of any comparative study.

## Toward an evolutionary framework for understanding primate brain organization

We have illustrated how MRI-based methods can provide comparative neuroscience with a wealth of data on the organization of different primate species' brains and discussed methods to analyse these data to make meaningful statements about similarities and differences between brains (Figure 1, first four columns). However, a full understanding of primate brain evolution needs to move beyond mere descriptions of difference between brains in two ways. First, these statements about brain *structure* needs to linked to behavioral data in order to allow inferences about brain *function* (Figure 1, right column). Second, when comparing data from different species, this should be done in a proper analytical framework to deal with the statistical challenges of non-independent data. In this section, we briefly discuss these two challenges. Since most studies employing a comparative MRI-based approach to date have focused on a two or three species, most of these methods are still under development.

### Theories of primate brain evolution and behavioral data

The ultimate goal of the neuroecological approach to primate brain organization is to understand how each brain is adapted to the environment of its owner. Fortunately, there is a large and detailed literature characterizing the ecological niches and behaviors of virtually every primate species (e.g., Smuts et al., 1987; Strier, 2007). This work has produced insights and hypotheses into primate behavioral evolution. A complete review of this work is outside the scope of this manuscript, but we will highlight some aspects relevant to the current discussion. Two classes of theories have dominated the field of primate evolution and provide the most common explanations for the large brain size of primates and its the variability within the primate order: those related to the animals' foraging ecology and those related to the complexity of their social life. The various theories differ in their view as to what behavior has most impact on an animal's fitness (Dunbar, 2010).

Foraging hypotheses emphasize the challenges faced by primates' particular diet. According to one view, early primates are thought to have evolved to exploit a niche in the small branches of trees, feeding on insects, nectar and flowers (Sussman, 1991; Bloch and Boyer, 2002). Such resources, while rich in calories, present unique foraging challenges in terms of their distribution, the volatility in their availability, the predation risks associated with foraging activity, and the sensory-motor mechanisms required to identify, manipulate, and extract nutritious parts (Ross, 2000). Dealing with these challenges is argued to have led to major changes in the size and organization of the primate brain. Clutton-Brock and Harvey (1980), for example, showed that primate brain size correlates with diet and home range across species and this has been invoked to explain the fact that, for example, the predominantly leaf-eating howler monkeys have a smaller home range and a relatively smaller brain than the predominantly fruit-eating spider monkeys (Milton, 1988): individual species of fruits are more distributed and available for a shorter periods than are leaves, thus requiring larger home ranges as well as the ability to predict when food patches can be found. Recently, Passingham and Wise discussed at book-length how the evolution of prefrontal cortex in the primate lineage may be related to addressing foraging challenges (Passingham and Wise, 2012).

The second major class of explanations relates brain size to social abilities. Primate species differ strongly in their social behavior, and there seems to have been a general increase in the complexity of primates' social life over the course of evolution (Shultz et al., 2011). In living primates, a more complex social life, whether indexed by group size, grooming clique size, the frequency of coalitions, or the frequency of tactical deception, correlates with a relatively larger neocortex (Dunbar and Shultz, 2007a). Hypotheses focusing on social behavior have changed substantially over time. The original Machiavellian hypothesis (Byrne and Whiten, 1988) claimed that the more social species of primates competed amongst themselves in more complex ways for access to important resources (food, mates, etc.) and that it was the cognitive demands imposed by this complex form of sociality that required a larger brain. In contrast, recent versions of the “social brain hypothesis” emphasize the demands of coordinating behavior with other group members and social bonding as the crucial challenge to living in larger groups, with coordinated groups being an essential intermediate step that allows an ecological problem (either more efficient foraging or defense against predators or conspecific raiders) to be solved (Dunbar and Shultz, 2007b; Dunbar, 2010).

**Figure 3:**
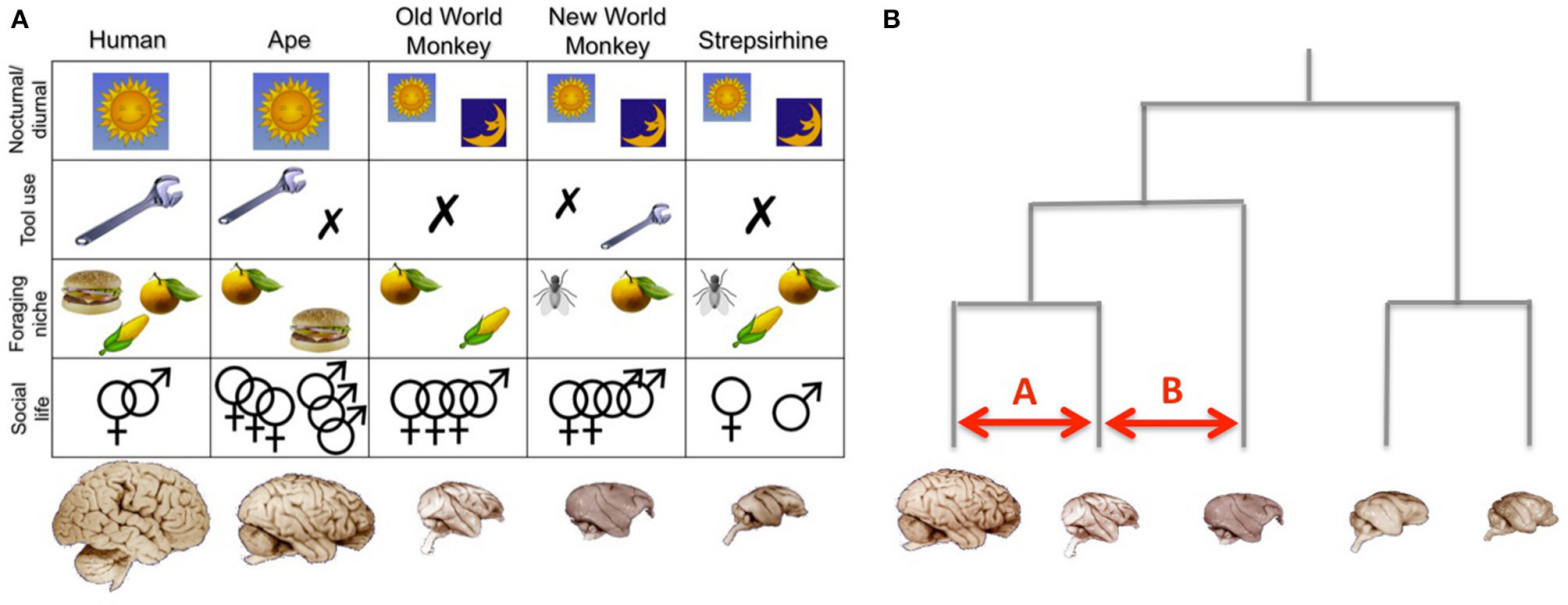
**Analysing the relationschip between brains and behaviors**. **(A)** In a multivariate comparative approach, each brain is viewed as a unique combination of variables, including whether the animal is active during night or day, whether it uses tools regularly, occassionally, or not at all, its diet, and the complexity of its social life. By using a whole-brain and multi-variate approach it is possible to investigate how differences in specific aspect of brain organization are related to different ecological variables. Note that this figure merely represents an idealized illustration. **(B)** Phylogenetic analyses take into account the evolutionary relationships between the studied species. In this context comparisons *A* and *B* are not equivalent, since they involve animals that share a different phylogenetic relationship. Note that this figure merely represents and idealized illustration, not an actual phylogenetic tree. Brain images are reproduced from the University of Wisconsin and Michigan State Comparative Mammalian Brain Collections and the National Museum of Health and Medicine (www.brainmuseum.org); preparation of these images was funded by the National Science Foundation and National Institutes of Health.

Distinguishing between these hypotheses faces the problem that different aspects of a species' niche are often correlated. Some recent work has therefore argued against a too modular view of primate intelligence (Reader et al., 2011) and warned against single monolithic explanations for the evolution of large brains (Barton, 2012). Care needs to be exercised in identifying the correct level of explanation (functions, mechanisms, systemic constraints, emergent consequences, etc.) because what look like alternative hypotheses may actually be different explanatory levels within the same explanatory system (Dunbar, 2010). Group living, for example, may not be an alternative to efficient food finding as an explanation for large brains, but instead be the intermediate mechanism that allows foraging to be done efficiently. The issue here is whether efficient foraging arises from individual trial-and-error learning or as a consequence of a social process, and, secondarily, whether these even involve different kinds of cognition (and hence neural networks). In addition, biological explanations commonly involve complex feedback loops (Dunbar and Shultz, 2007b). Because most species differ in multiple facets of behavior, a comparison between a very limited number of species is often confounded by multiple ecological and behavioral differences. Using a larger sample of species, in which the various behaviors vary sufficiently, allows one to address these problems statistically. Such a “general linear model” approach (Figure 3A) is basically an extension of the more traditional comparative studies, focusing on variance explained by one behavioral variable while taking into account variance in another. However, this is only the beginning of a much larger effort required to deal with the multiple interacting variables under study in this field.

The availability of the types of neuroanatomical data that we have discussed in this paper might dovetail with such a multivariate approach to primate behavioral data. Rather than correlating a global variable such as relative brain size with multiple behavioral variables, it is possible to relate distinct aspects of brain organization to specific differences in behavior. However, when attempting such an approach it is vital to consider whether all species studied can compared in the same way.

### Analysing comparative data in an evolutionary framework

Because species share similarities due to common ancestry as well as convergent evolution, individual primate species cannot be treated as phylogenetically independent data points. Accordingly, valid statistical inferences about adaptive evolution require quantification of independent evolutionary change. Comparing two closely related animals (comparison A in Figure 3B) is not the same as comparing more distantly related animals (comparison B in Figure 3B). This problem has long been recognized in evolutionary biology and a range of phylogenetic analysis techniques are available to deal with such dependencies (Harvey and Pagel, 1991; Nunn, 2011). A detailed explanation of these techniques is beyond the scope of this paper, but in brief they use evolutionary trees to account for uncertainty in the estimates of phylogenetic relationships so as to control for common ancestry. Such methods have been used by biologists to examine a wide range of evolutionary issues, for example to reconstruct the behavior of ancestral species, to locate transitions in genetic or phenotypic traits on the phylogenetic tree and to examine the correlated evolution of traits so as to test specific evolutionary hypotheses (see Harvey and Pagel, 1991; Nunn, 2011).

Phylogenetic analyses investigating between-species differences while taking into account evolutionary history have been applied to neuroanatomical data (Shultz and Dunbar, 2010; Reader et al., 2011; Smaers et al., 2011; Barton, 2012), but these studies have mostly focused on variations of the size of large subsections of the brain. They have, to our knowledge, not yet been applied to the data types discussed in the current manuscript. For instance, all studies identifying changes in connectivity rely on direct comparisons between a small number of species. The phylogenetic framework will allow a much better assessment of whether any observed differences indeed reflect adaptations in a particular species or lineage, and will allow researchers to determine the relationships between variation in connectivity and other anatomical variables such as volume and number of neurons within connected brain regions. Moreover, these techniques allow one to formally compare the evolution of behavioral and neural traits, providing stronger evidence that any structural adaptation is related to a behavioral, i.e., functional, adaption.

## Conclusion

We have argued that recent advances in MRI data acquisition and analysis methods provide comparative neuroscience with new opportunities for studying between-species differences in brain organization in a much wider range of species than ever before. While we do not mean to suggest that these methods should—or indeed can—replace traditional neuroanatomical techniques, they do provide the potential for previously unobtainable insights. Moreover, we have argued that this will allow comparative neuroanatomical data to be related to a much wider variety of behavioral data and analyzed in a proper phylogenetic framework. Only then can we understand how each primate species' brain supports a unique behavioral repertoire, adapted throughout an idiosyncatic evolutionary history to suit a particular ecological niche. This endeavor can lead to a better understanding of individual primate species' brains, including that of the human primate, but might also yield more general insight into evolutionary principles (cf. Striedter, 2005) and clinically relevant translational insights (cf. Kalin and Shelton, 2003).

### Conflict of interest statement

The authors declare that the research was conducted in the absence of any commercial or financial relationships that could be construed as a potential conflict of interest.
